# Latent by-product of substance use: Burden of care

**DOI:** 10.1192/j.eurpsy.2021.1490

**Published:** 2021-08-13

**Authors:** A. Channa, H. Mohsin

**Affiliations:** 1 Psychiatry, Heath Lane Hospital, Birmingham, United Kingdom; 2 Liaison Psychiatry, St. Vincent’s hospital, Dublin, Ireland

**Keywords:** Caregiver burden, SURP, Caregiver mental health, Substance use

## Abstract

**Introduction:**

Substance use affects it’s user and also risks the health of the caregivers.

**Objectives:**

Identify persons at risk of developing substance use disorder, assess the burden borne by the caregivers and development of psychiatric illness.

**Methods:**

Clinical assessment based on DSM-V criteria was performed for SUD diagnosis. Data was recorded using Substance use risk profile scale (SURPs) on the patient and the caregivers were evaluated using M.I.N.I. International Neuropsychiatric Interview (M.I.N.I) and caregiver’s strain index (CSI).

**Results:**

81 participants-96% were male, mean age 32.4 years, 53.1% married, 72.8% employed and 52% lived in joint family system). The substance use ascertained were alcohol 24.7%, benzodiazepines 21%, cannabis 34.6%, opioid 30.9% and others 4.8%. 50% had substance use lasting 2-9 years. 50.6% reported starting as a recreation and the perpetuating factor for 49.4%. was emotional distress. 44% quit due to family pressure. On SURP, 85.2% demonstrated anxiety sensitivity, 96.3% were hopeful, 66% sensation seeking and 77% were impulsive. Caregiver mean age was 37.8 years, with two-third being parents and spouses. The burden reported was sleep disturbance 59.3%, inconvenience (61.7%) physical strain 46.9%, confining 50.6%, family adjustment 76.5%, plan changes 65.4%, emotional adjustment 88.9%, behavioral adjustment 74.1%, financial strain 80.2%, work adjustment 46.9%, 71.6% felt overwhelmed and 67.9% were upset about the changes from former self. Major depressive disorder was identified in 51.9% of the caregivers.

**Conclusions:**

**Figure:**
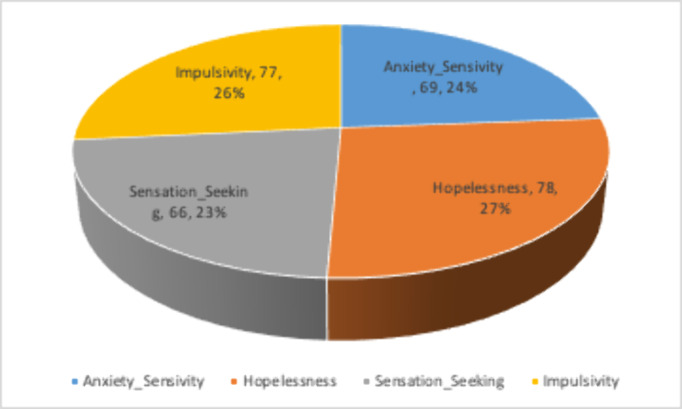

**Figure:**
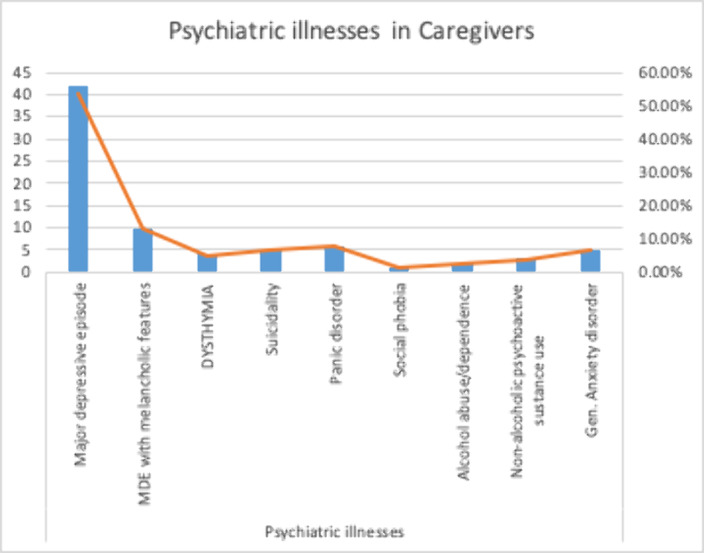

SURP identified personality features linked with risk of developing substance use disorder. The study also provided evidence for significant burden on caregivers and an increased likelihood to develop a psychiatric disorder.

